# A network analysis of executive functions before and after computerized cognitive training in children and adolescents

**DOI:** 10.1038/s41598-022-17695-x

**Published:** 2022-08-29

**Authors:** Iris Menu, Gabriela Rezende, Lorna Le Stanc, Grégoire Borst, Arnaud Cachia

**Affiliations:** 1 Laboratoire de Psychologie du Développement et de l’Education, UMR CNRS 8240, Universite Paris Cité, Paris, France; 2 Institut Universitaire de France, Paris, France; 3 Imaging Biomarkers for Brain Development and Disorders, UMR INSERM 1266, GHU Paris psychiatrie & neurosciences, Universite Paris Cité, 75005 Paris, France

**Keywords:** Human behaviour, Cognitive control

## Abstract

Executive functions (EFs) play a key role in cognitive and socioemotional development. Factor analyses have revealed an age dependent structure of EFs spanning from a single common factor in early childhood to three factors in adults corresponding to inhibitory control (IC), switching and updating. IC performances change not only with age but also with cognitive training. Surprisingly, few studies have investigated training-related changes in EFs structure. We used the regularized partial correlation network model to analyze EFs structure in 137 typically developing children (9–10 years) and adolescents (15–17 years) before and after computerized cognitive training. Network models (NMs) —a graph theory-based approach allowing us to describe the structure of complex systems— can provide a priori free insight into EFs structures. We tested the hypothesis that training-related changes may mimic developmental-related changes. Quantitative and qualitative changes were detected in the EFs network structure with age and also with cognitive training. Of note, the EFs network structure in children after training was more similar to adolescents’ networks than before training. This study provided the first evidence of structural changes in EFs that are age and training-dependent and supports the hypothesis that training could accelerate the development of some structural aspects of EFs. Due to the sample size, these findings should be considered preliminary before replication in independent larger samples.

## Introduction

Executive functions (EFs) are a set of high-level cognitive functions that allow an individual to intentionally regulate his or her thinking and act to achieve goals^1^. Three EFs are commonly distinguished: inhibitory control, working memory or updating, and cognitive flexibility or switching^2^. These functions are necessary for the development of more complex skills such as reasoning^3^, theory of mind^4–6^, arithmetic^7–9^, decision-making^10^ and creativity^11,12^. EF abilities improve with age^13–19^ under strong genetic and environmental influences^2,20–23^.

Studies of individual differences in EFs indicate that performance on tasks designed to tap a specific EF domain (e.g., inhibitory control) is correlated with but also separable from, performance on tasks tapping other EF domains (e.g., switching)^24^. Moreover, although scores on various EF tasks are often positively correlated with each other, these correlations are often not higher than those between EF and non-EF tasks. In this context, studies have investigated the structure of these functions to determine the extent to which (a) they reflect distinct or common abilities and (b) these abilities become more specific with age. Using structural equation modeling (SEM), Miyake et al.^1^ proposed a hierarchical structure of EFs, with three latent factors representing each EF domain. In adults, these latent factors are separable (EF diversity), although they share a significant proportion of variances (EF unity, or common-EF ability)^20^. The EF structure evolves from a one-factor structure in early childhood with no clear separation among EF tasks^25–28^ to a two- to four-factor structure in adolescence^15,21,28–31^. Of note, some studies also report an organization with more than one factor in young children^31^ and fewer than three factors in older children^32^. A recent meta-analysis^33^ tested seven models of EF structure and found some evidence for greater unidimensionality of EFs among child/adolescent samples and both unity and diversity among adult samples. The developmental organization of EFs^15^ is supported by a recent brain imaging study reporting an increasing segregation of structural brain network modules with age, and this segregation mediates the effects of age on EFs^34^. In addition, a recent behavioral study on children from 7 to 15 years^24^ found that age mostly impacts the common EF loadings of inhibitory control and switching. Hence, while in childhood, updating, switching and inhibitory control likely rely on similar underlying cognitive processes, in adolescence, EFs become more specialized and independent.

Because EFs are implicated in learning, academic achievement, psychiatric health, and everyday functioning^2,35^, several intervention programs have tested the possibility of stimulating various aspects of EFs, including working memory^36,37^ and inhibitory control (hereafter referred to as IC)^2,10,38–41^. These studies, conducted in children, adolescents, and young adults, have shown that it is possible to train EFs and have raised the question of the duration of the effects of training^39^ as well as the possibility of transferring the effects of training an EF to other executive or cognitive domains^10,39–41^. Indeed, most training studies aimed to determine to what extent executive control training and IC training in particular transfer to untrained tasks within the same domain or EF (i.e., near transfer) or in other domains or functions (i.e., far transfer). While some studies reported near and far transfer effect in preschoolers (for near transfer: Zhao et al.^41^ and for far transfer: Liu et al.^40^; Rueda et al.^42^) other studies have shown no near or far transfer effects of IC training^43–45^. Importantly, to date no EF training studies have assessed the extent to which EF training changes the structure of EFs.

Another way to understand EF organization and how it changes with age is to use network modeling (NM), a graph theory based-approach allowing us to describe the structure of complex systems^46^. The underlying principle of NM is that systems can be represented as nodes that are interconnected with edges (the thicker the edges are, the stronger the interconnection). The complete graph (nodes and edges) summarizes the pattern of relations among the elements^47^. While in SEM, shared variance of observed variables (e.g., scores on cognitive tasks) is assumed to reflect a latent construct (e.g. inhibitory control or working memory), in NM, shared variance is assumed to reflect a causal network^48^.

NM applied to EFs allows us to identify which nodes (here, a specific EF task) play a pivotal role within the whole network (here, different EF tasks). In addition, NM has the potential to test theoretical models on how EF structures transform with age and more specifically which components can become more central to general executive processing and, therefore, have a greater influence on other EF processes with age. Using NM on a twin cohort aged 7 to 15 years of age, Hartung et al.^24^ found that the interconnections between EF tasks remained stable with age except for the inhibition tasks, whose shared variance with the other EF tasks was reduced with age. These findings provided convergent evidence that IC is particularly important for allowing young children to employ other EFs in pursuit of goals but plays a lesser role in regulating other EFs later in development^32,35,49^. NM can also provide interesting insights into the effects of training on the structure of cognitive functions. To date, only one study has used NM to treat such effects in young adults^50^. The study showed that the interconnections between 25 variables related to mindfulness, compassion, psychological well-being, psychological distress and emotional-cognitive control changed after a mindfulness-based stress reduction (MBSR) program.

**Figure 1:**
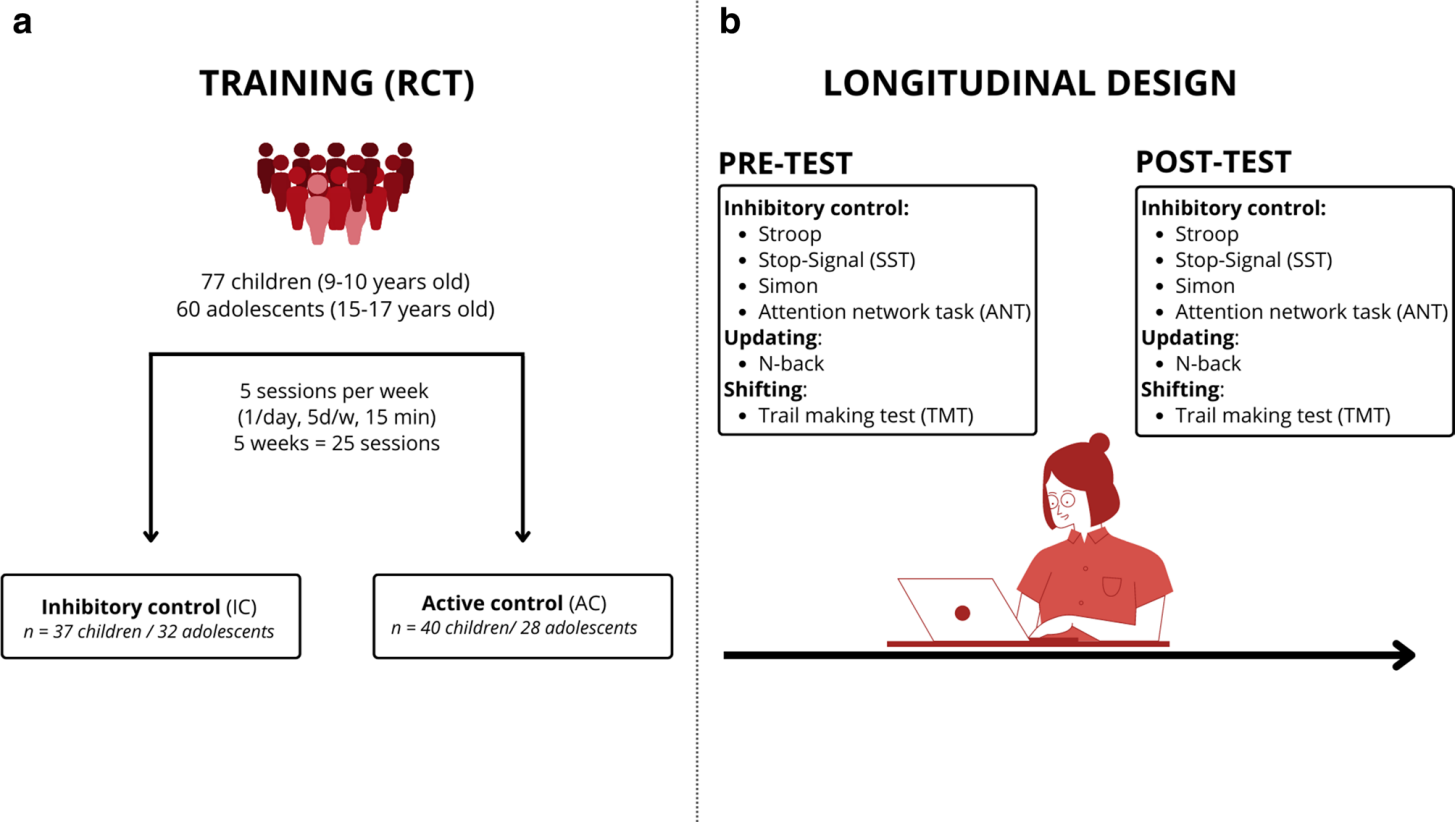
Experiment design. (**a**) 77 children (9–10 y.o.) and 60 adolescents (15–17 y.o.) were asked to perform inhibitory control tasks (IC group) or knowledge- and vocabulary-based tasks (Active Control (AC) group) 15 min per day, 5 days a week for 5 weeks (25 sessions) on a tactile tablet. Participants were randomly assigned to the IC or the AC groups as in a Randomized Controlled Trial (RCT). The AC condition allowed to control for test–retest effects. In both IC and AC training, the difficulty was progressively increased and adapted in real time to the learning curve of each participant to maintain the motivation of the participant and to prevent automaticity. (**b**) Participants performed a cognitive battery in the pre- and post-training sessions (longitudinal design) measuring different facets of executive functions: cognitive flexibility/switching (TMT), working memory updating (N-back task), and inhibitory control (Stroop task for interference control, Stop Signal and Simon tasks for response inhibition and ANT for attentional inhibition).

In the present study, we investigated how the structure of EFs was affected by training EFs in children and in adolescents using NM. By using such an approach, we aimed to determine whether training speeds up the development of EFs or qualitatively changes the development of EFs by deviating from the developmental trajectory typically observed from childhood to adolescence^51^. To this end, we assessed EFs in 77 children (9–10 years) and 60 adolescents (16–17 years) before and after 5 weeks of computerized training. Children and adolescents were randomly assigned to an IC training group or an active control group (Fig. 1). We reasoned that if training IC speeds up the development of IC, then training-related changes should mimic developmental-related changes, namely, the structure of EFs in children after IC training but not after control training should be more similar to the structure of EFs in adolescence than before training. On the other hand, if training IC changes the developmental trajectories of IC, then the structure of EFs in children after IC training should differ from that before training (but not after control training) and from the structure of EFs in adolescence. This study, which is preliminary given the sample size, will allow testing these hypotheses before replication in an independent sample.

## Results

We investigated the effects of development and cognitive training on EFs using a 6-node network with 4 measures taping on different aspects of IC (Stroop on interference control, Stop Signal and Simon on response inhibition and Attentional Network Task or ANT on attentional inhibition), 1 measure of switching (Trail making test or TMT) and 1 measure of updating (N-back) was constructed and estimated.

### Developmental analysis: children vs. adolescents at pretest (before training)

**Figure 2:**
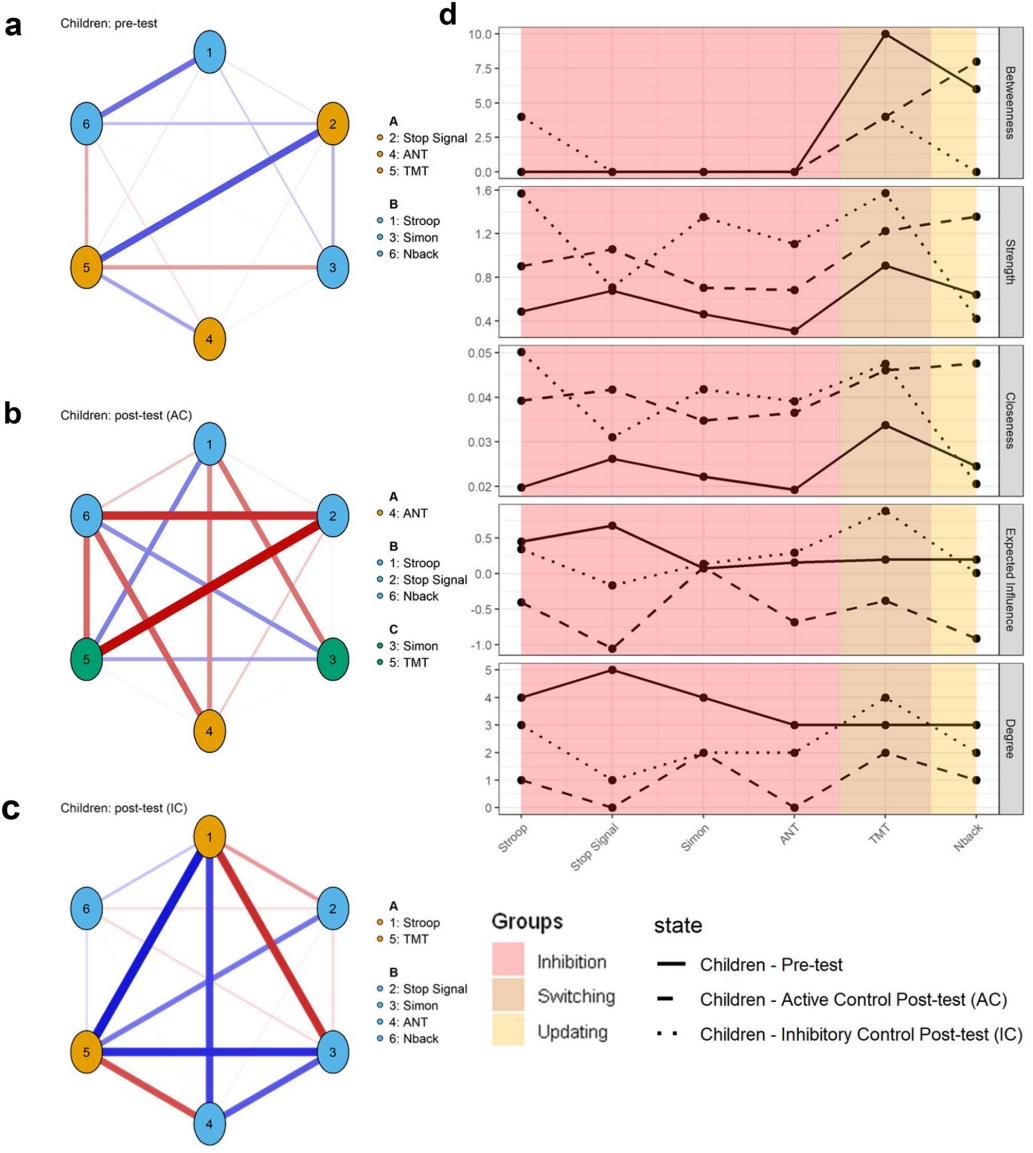
Six-nodes networks for children, before (**a**), after active control training (AC group; **b**) and after inhibitory control (IC group; **c**) training with the corresponding centrality indices (**d**). Color of nodes correspond to the communities. Each number in the networks corresponds to an EF task (see details in the legend).

**Figure 3:**
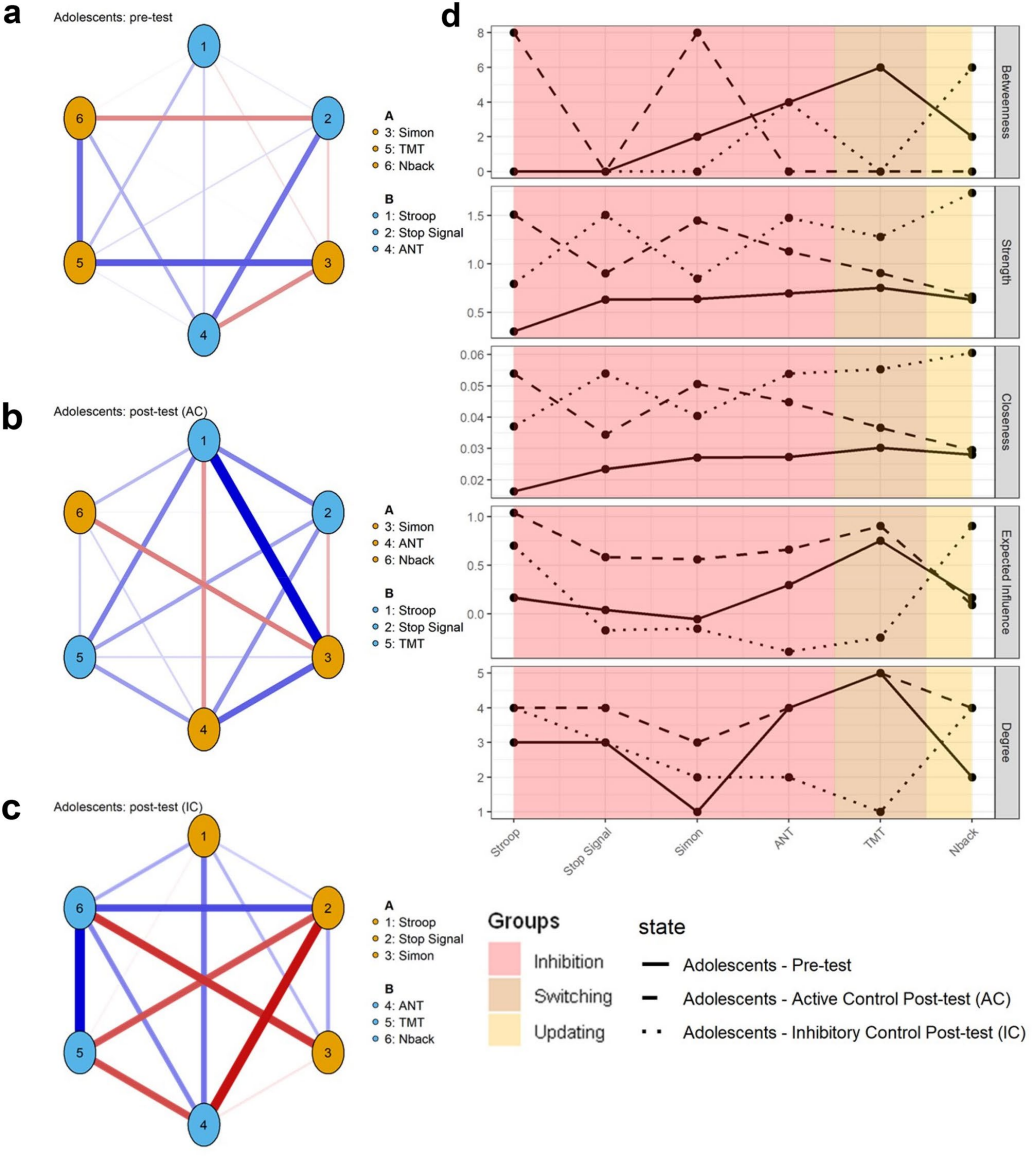
Six-nodes networks for adolescents, before (**a**), after active control training (AC group; **b**) and after inhibitory control (IC group; **c**) training with the corresponding centrality indices (**d**). Color of nodes correspond to the communities. Each number in the networks corresponds to an EF task (see details in the legend).

We first studied the EF structure in children and adolescents from the analysis of the EF networks at pretest before training (see Figs. 2 and 3). Visual inspections indicated that networks in adolescents present more and stronger connections than in children.

This visual inspection was followed by a quantitative analysis of the network topology using classical graph indices (see Figs. 2 and 3). The different indices were similar in children and adolescents. Three common centrality measures were used to quantitatively characterize the network at node levels^52^: strength (a measure of how strongly a node is directly connected with the network), betweenness centrality (a measure of how a node is central in connecting other variables) and closeness centrality (a measure of how strongly a node is connected indirectly with the network). Higher closeness centrality indicates that a node (task) is more related, even indirectly, to other nodes (tasks). Higher strength indicates that a node(task) is more strongly connected to other nodes (tasks). Because these indices are calculated based on the absolute values of edge-weights and may therefore miss information on the network structure if negative relationships between nodes are present^53^, two other centrality measures were also used: expected influence (EI), which is the sum of both positive and negative weights between a node and all the other nodes in the network, and degree, which is the number of connections for each node in the network, thus defining hubs (nodes with highest degree). In both children and adolescents, the variables with the highest betweenness were also the variables with the highest strength, closeness and EI. However, such central variables varied with age: in children, the most central nodes included the Stroop, Stop Signal and TMT while in adolescents, they included the ANT and TMT. In children, a high number of relations (i.e., high degree) was generally accompanied by low weights (i.e., low EI and strength).

In adolescents, analysis of hubs revealed homogeneous results, with similar weights over the four indices. Overall, these indices were slightly lower in children than in adolescents, reflecting a less connected network in 9–10-year-old children than in 16–17-year-old children.

We then analyzed the communities. A community corresponds to a set of nodes that cluster more strongly among each other than with other nodes in the network; such communities reflect high mutual influences among nodes in a given cluster. The community analysis detected two clusters in each age group’s network (Figs. 2 and 3A–C). In children, the two clusters were as follows: (1) Cluster A (in orange), which included 3 nodes corresponding to executive functioning (Stop Signal, ANT and TMT); (2) Cluster B (in blue), which included 3 nodes corresponding to IC and updating (Stroop, Simon and Nback). In adolescents, the two clusters were as follows: (1) Cluster A (in orange), including 3 nodes corresponding to executive functioning (Simon, TMT and Nback); (2) Cluster B (in blue), including 3 nodes corresponding to IC (Stroop, Stop Signal and ANT). The only difference between children and adolescents’ networks was the cluster switch of Stroop and TMT. Finally, the negative correlation between edge weights across networks (r = − 0.51) reflected the differences previously observed in network connectivity.

### Training effects: pretest vs. posttest

**Figure 4:**
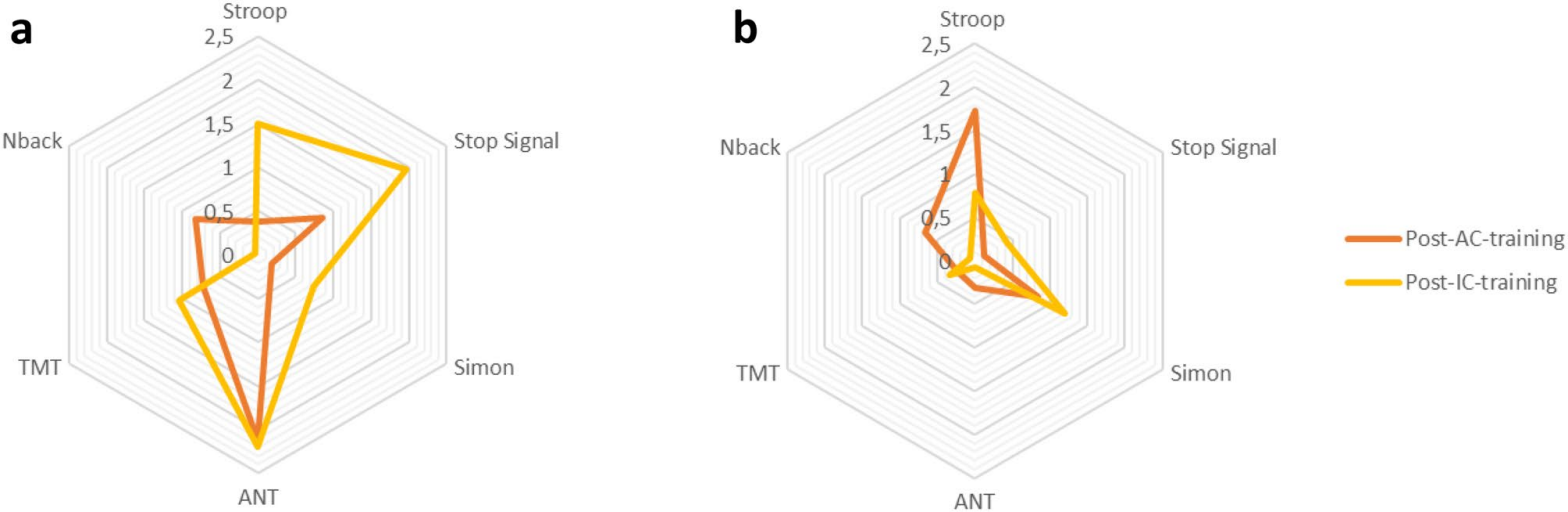
Relative changes in EF tasks after cognitive training in children (**a**) and adolescents (**b**). The radar graphs represent relative changes after inhibitory control (IC, in yellow) and active control (AC, in orange) training. Values correspond to − log(p), with p being the main effect of training in the repeated measures ANOVAs presented in Table 1.

The changes in EF structure were first investigated using classical univariate repeated-measures ANOVAs applied to the two age groups and the two training groups separately (Table 1 and Fig. 4). These analyses detected a significant change in the Stop Signal (p < 0.05) and Stroop (p < 0.05) along with a marginal change in TMT (p = 0.09) for children after IC training. In adolescents, a significant change in the Stop Signal was detected following AC training (p < 0.05). All the other analyses failed to reach significance (all p values > 0.14).

**Table 1:** Efficiency of EFs in children and adolescents before and after an active control (AC) or an inhibitory control (IC) training. For all tasks, scores were derived from RTs (in s).

	Pre IC-training	Children			Pre IC-training	Adolescents		
		Pre AC-training	Post IC-training	Post AC-training		Pre AC-training	Post IC-training	Post AC-training
	Mean ± SD	Mean ± SD	Mean ± SD	Mean ± SD	Mean ± SD	Mean ± SD	Mean ± SD	Mean ± SD
SST	0.26 ± 0.08	0.25 ± 0.08	0.21 ± 0.06*	0.23 ± 0.08	0.16 ± 0.03	0.16 ± 0.03	0.15 ± 0.04	0.16 ± 0.03
Stroop	0.11 ± 0.16	0.13 ± 0.15	0.06 ± 0.10*	0.08 ± 0.16	0.07 ± 0.09	0.09 ± 0.08	0.04 ± 0.05	0.06 ± 0.07*
Simon	0.04 ± 0.03	0.04 ± 0.03	0.03 ± 0.03	0.03 ± 0.04	0.08 ± 0.05	0.08 ± 0.06	**0.02 ± 0.02**○	0.02 ± 0.02
ANT	0.04 ± 0.03	0.03 ± 0.03	0.06 ± 0.04**	0.05 ± 0.04**	0.04 ± 0.02	0.05 ± 0.02	0.04 ± 0.02	0.04 ± 0.03
TMT	22.14 ± 12.01	18.18 ± 10.04	**16.40 ± 9.07**○	15.03 ± 6.66	6.87 ± 3.63	9.62 ± 4.73	7.83 ± 4.33	8.71 ± 4.15
N-back	0.09 ± 0.23	0.10 ± 0.20	0.06 ± 0.23	0.12 ± 0.24	0.04 ± 0.07	0.06 ± 0.08	0.07 ± 0.11	0.09 ± 0.11

*SST* stop signal task, *TMT* trail making test.

Training-related changes in task efficiency were evaluated with repeated measures ANOVAs. Significance levels: ^○^ < 0.10; *< 0.05; ** < 0.01; *** < 0.001.

Complementary analyses, including age and training groups as factors in order to investigate possible age- and training-specific effects, only revealed significant main effects of the age group for SST (p = 1.3 × 10^–5^) and for TMT (p = 0.01) but no interaction effects involving the age nor the training group (all ps > 0.27; see details of the analyses in Table S1). Post-hoc analyses, with Tukey correction for multiple testing, revealed significant pre-post changes in children in IC group for SST (p = 0.009) and TMT (p = 0.03).

Of note, Welch Two Sample t-tests revealed no significant differences between the two training groups at pretest except for TMT in adolescents (t (39.39) = − 2.16, p < 0.05) where adolescents affected to the IC training showed lower score (6.87 ± 3.63) than those who were affected to the AC training (9.62 ± 4.73). All other ps > 0.17. See details of raw pretraining and posttraining scores for the three EF tasks in Table 1 and in the radar-plots representing the relative changes after the two types of training in Fig. 4.

**Figure 5:**
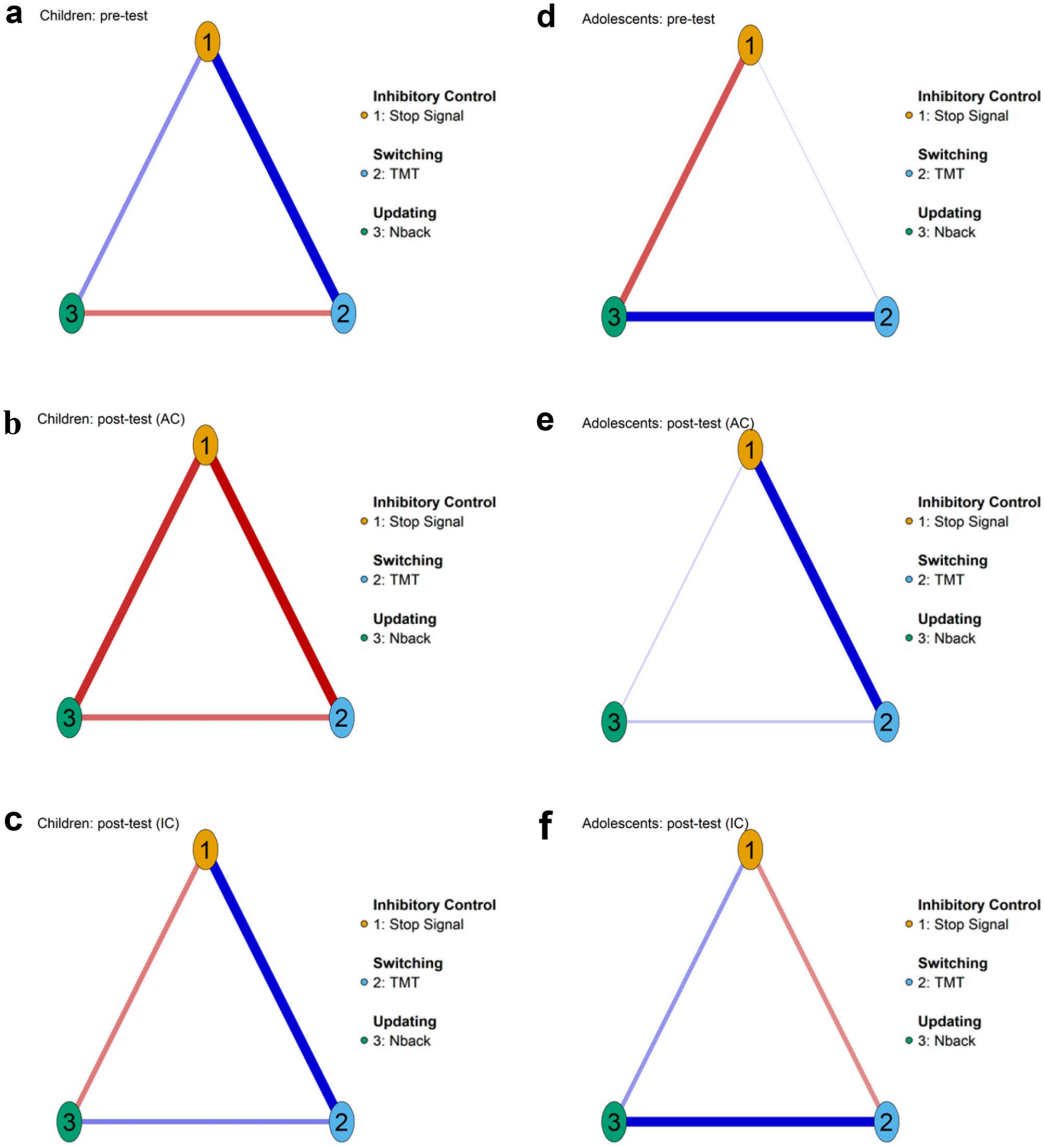
Three-nodes networks for children (**a**–**c**) and adolescents (**d**–**f**), before (**a**,**d**), after active control training (AC; **b**,**e**) and after inhibitory control (IC; **c**,**f**) training. Color of nodes correspond to an executive function. Each number in the networks corresponds to an EF task (see details in the legend).

These standard analyses were further investigated by comparing the network structure in the pretest and posttest for children (Figs. 2 and 5) and adolescents (Figs. 3 and 5).

### Cognitive training in children

The children’s 6-nodes-network had a different organization, with denser, more numerous and stronger connections after training compared to pretest, especially after the IC training. Almost all variables showed increasing strength (except for the Stop Signal task) and closeness (Fig. 2B). Stroop and TMT remained the most central nodes of the network after IC training. After AC training, most of the variables increased in strength and closeness, and N-back became the most central node in the network along with TMT. The centrality indices revealed poor connections at pretest in children (strength < 1 and closeness < 0.03) while at posttest, strength and closeness increased, but differently after AC and IC training.

The community analysis (Fig. 2) detected two communities both pretest and after IC training and three communities after an AC training. After AC training, the three clusters were as follows: (1) Cluster A (in orange), including only ANT; Cluster B (in blue), including Stroop, Stop Signal and N-back and Cluster C (in green), including Simon and TMT. After IC training, the two clusters were: (1) Cluster 1 (in orange), including Stroop and TMT and Cluster B (in blue), including Stop Signal, Simon, ANT and Nback.

Correlations between edge weights across networks were low both after AC (rAC = − 0.30) and IC (rIC = − 0.23) training indicating few similarities between networks before and after training.

### Cognitive training in adolescents

In adolescents (Fig. 3), the differences in network structure between pretest and after IC training are less important than in children (see Fig. 3a–c). Nevertheless, after IC training, almost all variables except Simon increased in strength and closeness. Analysis of EI and Degree highlights the centrality of Stroop and Nback, which have the highest scores in these indices. After AC training, fewer changes occurred with an increase in Stop Signal and Simon for both Strength and Closeness. However, these changes were less important than those after IC training.

Community analysis revealed small cluster changes after training, with two nodes being switched after AC training (TMT and ANT) and after IC training (ANT and Simon; see details in Fig. 3). As for centrality indices, changes in communities were less important in adolescents than in children.

Correlations between edge weights across networks between pretest and posttest were low in both AC (rAC = − 0.08) and IC training (rIC = − 0.11), supporting very few similarities between networks before and after training.

In addition to the 6-node networks, a complementary analysis of balanced 3-node networks, where each node represented an EF (Stop Signal for IC, TMT for switching and N-back for updating; Fig. 5), was performed. The Stop Signal, the IC task with the most significant progression after training (see pre-post changes in Table 1), was selected as IC measure. This analysis provided similar results to the previous analysis obtained with the 6-node network, namely greater network connections in childhood than in adolescence and similar network changes related to training and to development.

## Discussion

In this study, we report the first NM analysis of EF structure changes with age and cognitive training. Based on the hypothesis that training mimics development and can therefore accelerate cognitive changes with age, we anticipated a switch from a centralized to a distributed EF network from childhood to adolescence^1,34^ as well as in children after IC but not AC training.

Quantitative and qualitative differences were detected in the EF network structure between children (9–10 years) and adolescents (16–17 years). The increased connections with age between children’s and adolescents’ networks between tasks tapping different EF domains support the previously reported increasing shared variance among EF variables during development^28^. This increased number of connections was confirmed by an increased overall centrality in adolescents compared to children. These findings are also consistent with a study on the development of EF structures from 7 to 15 years of age reporting increased centrality indices (closeness and strength) for EF tasks after 13 years of age^24^. Our results also support a recent cross-sectional study using network analysis to examine changes in EF organization from 3 to 85 years of age^54^. This study reported an increase in inter-EF connections (increasing strength and expected influence) from 15.5 years of age^54^, consistently with our findings. However, this study also demonstrated that this increase was preceded by a decrease in the centrality indices from early childhood. Our two age groups are thus just around the point of inversion, it might thus be interesting in the future to extend our analyses with participants just at the point of inversion. Moreover, an accelerated longitudinal design study also suggested organizational changes between ages 8 and 14, along with change for each age group within a single year^55^. Thus, it might be relevant to narrow the age range in order to investigate finer developmental changes. Analysis of centrality also revealed that Stroop, Stop Signal and TMT—the first two on IC and the last one on switching—are central in the EF network of children. This is consistent with previous studies that reported that IC is central for children to employ other EFs^32,35,49^. However, according to these studies, with age and EF development, IC becomes less central, while in adolescence and adulthood, working memory increases its role in regulating EFs^32,35,49^. We thus expected N-back to be the most central node in adolescents. Instead, TMT and ANT—tapping on switching and attentional IC—were the central nodes of the network. Nevertheless, it should be noted that N-back had a high centrality in adolescents’ graphs, and this centrality increased after 5 weeks of IC training, perhaps reflecting the increasing central role of working memory in EFs’ regulation. Moreover, these results are consistent with a recent study that emphasized the increasingly critical role of Switching during the development, which would act as a mediator between IC and Updating from adolescence^54^. On another hand, the community analyses revealed an organization of EFs in two clusters in both children (two clusters with mixed-EF tasks) and in adolescents (one cluster with IC tasks and a second cluster composed of three tasks measuring the three different EFs). Previous SEM studies reported a differentiation of EF organization between middle childhood and adolescence^15,21,24,28–31^. It is important to note that the clusters obtained with community analysis are determined a posteriori (via a data-driven approach), while the factors obtained in SEM analysis are determined a priori (via a hypothesis-driven approach). Indeed, clusters derived from community analysis correspond to nodes with high mutual influence and are therefore dependent upon the data under analysis, while SEM clusters correspond to latent factors that were defined before the analysis. Hence, taken together, previous SEM studies and current NM analyses, which are based on complementary approaches, converge toward changes in EF structure from childhood, with a more general composition of EFs, to adolescence, with more specified EFs.

In addition to developmental changes, quantitative and qualitative changes in the EF structure were also found after training one EF, namely, IC. The results showed that after IC training in children, networks have increasingly stronger connections both within and between EFs and are therefore more similar to adolescents’ networks than before training. On the other hand, in adolescents, changes in the EF network were subtler. More precisely, after AC training, the number of connections decreased, but some edges increased in weight, whereas after IC training, the edge weights became much more important, reflecting a more integrated network. However, these lower changes in adolescents compared to children may also be interpreted in terms of reduced training effects in adolescents. Complementary analyses using classical repeated-measures ANOVAs (see Table S1 and Fig. S1 in Supplementary Materials) indicated that adolescents did not improve their performances in the six EF tasks, while children improved their performances after IC training for Stop Signal, ANT and tendentially for TMT. The lack of progress in adolescents might reveal a ceiling effect (see Fig. S2 in Supplementary Materials). Of note, the AC and IC trainings were similar in children and adolescents, with difficulty adapted at the individual level. Importantly, except for TMT in children, classical repeated-measures ANOVAs did not detect important transfer effects, while NM could highlight changes in the organization of EFs, including both trained and nontrained tasks (e.g., Stroop and Stop Signal), thus revealing transfer effects. The community analyses also provided insights into training-related reorganizations of EF structures. In children, networks had two communities before and after the IC training and three communities after the AC training, whereas in adolescents, there were two communities before and after both types of training; these communities had only slight changes in composition, once again reflecting the reduced effects of training in adolescents compared to children. However, it can be noted that in adolescents, after IC training, one of the two communities was composed of only IC tasks and the other of attentional IC, switching and updating tasks, thus highlighting EF-specific effects.

The present study has several limitations that call for caution when interpreting the results. First, sample size is a critical issue for the reliability of statistical analysis, particularly for NM analysis^56^. Hence, despite the relatively large sample size used in this interventional longitudinal study in children and adolescents (N > 120), it is important to replicate the findings with confirmatory studies conducted on larger and independent samples. Of note, the recruitment and follow-up of typically developing school-aged children and adolescents enrolled in a 5-week longitudinal study with cognitive training on a tactile tablet raised sound logistical and practical issues which has constrained the sample size. Following recent recommendations for NM analysis with small sample size (i.e., approximately hundreds of participants), we limited the number of variables to 6^57^ as it allowed us to recover the global structure of the network even though the full network could not be measured. This criterion also led to an imbalance of the three EFs in the creation of the networks. Indeed, IC was overrepresented (4 nodes out of 6). As this was one being trained, it seemed important to look at the impact of training on the organization of this particular EF. Because such imbalance may bias the analysis, and particularly the estimation of the partial correlations, we completed our 6-node network analysis with a balanced 3-node network analysis with networks including one measure per EF. These 3-node network further confirmed the results provided by the 6-node network analysis, namely greater connections in childhood than in adolescence and similar network changes related to training and to development. A perspective is the inclusion of latent variables in the networks^58^, which could allow us to observe the links, without a priori, between tasks within the same EF latent variable. Second, the behavioral changes observed from the pre- to the post-training sessions might not be attributed only to the training per se but could also reflect a ‘regression to the mean’ effect^59^. This statistical phenomenon arises when a random variable—here task scores—is extreme at baseline but closer to the mean on follow-up or vice versa and typically affects longitudinal design such as the one used in the present study. However, it is unlikely that the difference in IC efficiency change from the pre- to the post-training sessions between the IC and AC training groups only reflects such a ‘regression to the mean’ effect because participants were randomly assigned to the two training groups, and thus, both groups were potentially equally affected by such an effect^60^.

NM provides an original and relevant way to investigate the effect of cognitive training on EF organization, complementary to more classical statistical approaches, such as univariate ANOVAs. Our study combining NM and classical ANOVAs appears to be relevant to analyses of developmental and training-related cognitive changes. Recent methodological developments, such as moderated network models^61^ or network model trees^62^, could be an interesting perspective to further explore factors that could influence network organization after an intervention. Because EF neural networks are known to vary with age and to correlate with EF behavioral performance^34^, a multimodal and multilevel approach combining network analysis at the behavioral level and the neural level (e.g., using resting-state functional magnetic resonance imaging)^63^, is likely an interesting direction to explore. Such an approach could provide a more complete view of EFs^64^ and could pave the way toward an integrative approach, including behavioral, neural and genetic and environmental levels.

## Methods

### Participants

We recruited 137 healthy participants from public schools: 77 children (33 males, M ± SD = 9.86 ± 0.55 years, range = 9–10 years) and 60 adolescents (20 males, M = 16.56 ± 0.48 years, range = 15–17 years). All participants were right-handed as determined by the Edinburgh Handedness Inventory^65^, were born full-term, had normal or corrected-to-normal vision, had no history of neurological disease and had no cerebral abnormalities. Parents or legal guardians gave written informed consent for the children and the adolescents, and all children and adolescents agreed to participate. All participants were tested in accordance with the national and international norms that govern the use of human research participants. The national ethics committee (Committee for the protection of persons, CPP) approved our study in children (ID-RCB 2015-A00383-46) and in adolescents (ID-RCB 2015-A00811-48).

### Cognitive training

In both IC and AC computerized 5-week training, the difficulty was progressively increased and adapted in real time to the learning curve of each participant to maintain his or her motivation and to prevent automaticity^36,66,67^. In each session, the level of difficulty was increased or decreased after each block of trials performed at a given level. All tasks were implemented on the tablet using E-prime 2.0.

The IC training included two tasks involving interference control (Color-Word Stroop task) and response inhibition (SST) because (a) IC is a multidimensional construct^2^ and (b) transfer effects can be potentially larger when the same cognitive function is trained with different tasks^66,67^. In particular, each task performed by the participants during the IC training involved different types of inhibitory control processes, namely response inhibition for the Stop-Signal task and interference control for the Color-Word Stroop task. The Stop-Signal task typically requires inhibiting a motor response action after it was initiated while the Color-Word Stroop task requires inhibiting task-irrelevant information (i.e., the color denoted by the word)^2^.

AC training consisted of knowledge- and vocabulary-based tasks of increasing difficulty^39^. On each task, 4-choice trivia-like questions were presented, and participants were asked to answer by pressing on one of the four answers presented on the screen. Participants were given a maximum of 30 s to reply to each question. An online pretest performed on more than 1600 children and adolescents helped us select the questions assigned to each of the 8 levels of difficulty. Ten questions were presented at each level.

At the end of each level, based on the achieved accuracy, participants earned points that they could redeem at the end of the 25 training sessions for a small gift (books and card or board games). In all four tasks, task difficulty was increased when participants achieved 90% accuracy at a given level, decreased when participants failed to achieve 70% accuracy at a given level and remained the same when participants reached accuracy between 70 and 90% at a given level. On each task of each training session, participants started the session with the difficulty level one level below the one they achieved in the previous training session. In the two IC training tasks, the difficulty of inhibiting either the motor response initiated or the irrelevant task information was adapted after each block of training. In the Stop-Signal task, by increasing the delay between the presentation of the stimulus and the presentation of the signal to stop, the inhibition of the motor response became increasingly more difficult^68^. In the Color-Word Stroop task, as the delay between the presentation of the word and the coloring of the word decreased, the inhibition of the task-irrelevant information (i.e., the color denoted by the word) became increasingly more difficult^69^. In the AC training tasks, the answers to the questions were increasingly more difficult to determine and were adapted after each block of training. At the end of each training session, parents were instructed to send the data file generated by E-prime 2.0, and participants were asked to complete a short autoevaluation of their motivation and engagement during the session. Levels reached at each training session are reported in Fig. S2 in the Supplementary Materials. Participants included in the analyses had to complete a minimum of 15 training sessions.

### Evaluation of EFs

Participants performed a cognitive battery in the pre- and post-training sessions measuring different facets of EFs. Six tasks were administered to measure the three EFs: cognitive flexibility (here referred to as switching), working memory updating (here referred to as updating), and inhibitory control. The task used to identify the switching factor was the trail making test (TMT)^70^ and for the updating factor, the N-back task^71^ was used. Four tasks were used to identify the inhibitory control factor: the Color-Word Stroop^72^, the Stop-Signal task^73^, the Simon task^74^ and the ANT (Attention Network Task)^75^; each of these tasks taps on different aspects of IC: Stroop on interference control, Stop Signal and Simon on response inhibition and ANT on attentional inhibition^2,76^. To limit potential differences in familiarity with the Stroop and Stop-Signal tasks used in the pre- and post-training sessions between participants of the IC and AC groups, we introduced a number of differences between these tasks and those used in the IC training sessions: (a) we used no control trials, and we did not vary the difficulty of the task in the Color-Word Stroop task; (b) the Stop-Signal delay was adapted on a trial-by-trial basis and not on a block-of-trial basis in the SST.

For each task, scores were screened and cleaned for possible aberrant values using a nonparametric approach: outliers were defined as values lower than median − 2.5 MAD or greater than median + 2.5 MAD (MAD: median absolute deviation) and considered missing values in the analyses.

### Construction and analysis of cognitive networks

NM were completed with classical univariate statistical analyses (Analyses Of Variances, ANOVAs). Like for NM, ANOVAs were conducted separately for each of the six EF tasks for the two age groups and the two training groups. In order to assess possible group-specific effects, complementary ANOVAs were run for each task, including age group (children vs adolescents) and training group (IC vs AC) in the models.

The repeated-measures ANOVAs were estimated using mixed-effects linear models. We used the package *lme4*^77^ with the Time (pre- or post-training) as fixed effects and intercepts for subjects as random effects. P-values were obtained by using likelihood ratio tests of the full model, including the tested effect against the model without the tested effect.

Then, separate networks, based on the correlation matrix of the EF task scores, were built for children and adolescents before training (pretest) and after training (inhibitory control training: posttest IC; active control training: posttest AC). Six networks (3 per age range) were estimated. These networks included 6 nodes corresponding to the scores of the 6 cognitive tasks, which we grouped into three EFs:

- Inhibitory control: Color-Word Stroop, Stop-Signal, Simon, and ANT scores (for Stop Signal, we calculated the SSRT as recommended^78^, and, for the other tasks, we calculated the difference in reaction time (RT) between incongruent and congruent trials)
- Switching: TMT flexibility score (RT difference between TMT-B and TMT-A)
- Updating: N-back score (RT difference between the 2-back and the 1-back trials).

The inclusion of trained (Stroop and Stop-Signal) and untrained (Simon and ANT) IC tasks allowed us to observe the direct effects of IC targeted training on the trained tasks but also on other IC tasks (intra-EF) and thus, to assess near transfer effects within the same EF. The inclusion of TMT and N-back allowed us to assess the effects of IC training on other EF tasks (inter-EFs) and thus to evaluate the effects of a more distant transfer while keeping the number of nodes limited to 6, as recommended for a sample size of approximately 100^57^.

In addition to the 6-node networks, balanced 3-node networks, with only one node per EF (Stop Signal for IC, TMT for switching and N-back for updating), were built. Such balanced networks overcome the issues for the interpretation of the 6-node network related to the partial network models that remove shared variance associated with all other EF tests in the model (i.e., the relationship between each inhibition task with switching and updating involved controlling for other tests of inhibition).

NM was used to analyze (1) the multiple relations (edges) between the different EF tasks (nodes) simultaneously and (2) how these relations change during development (children vs. adolescents) and after cognitive training (before and after training). We used the successive steps procedure proposed for network analysis in psychology^79^: (1) network estimation; (2) network inference (topological characterization); and (3) node community analysis. The interrelation between the different variables was modeled with a Gaussian graphical model (GGM^80^), a regularized partial correlation network (RPCN). The edge between two nodes/tasks corresponded to the partial correlation between the two corresponding variables, controlling for the effects of the remaining variables. We used Spearman correlations, as recommended^81^.

Statistical analyses were performed using R-statistical software, version 3.6.1 (R Development Core Team, 2014). The networks were constructed and visualized using the package *qgraph* version 1.6.4^82^. As previously recommended^83^, we investigated the robustness and replicability of the analyses (accuracy check) using the *bootnet* package version 1.4^81^. Figures of edge-weight accuracy can be found in the Supplementary Materials.

### Characterization of the networks

The networks were characterized using both quantitative and qualitative measures. Five classical centrality measures were used to quantitatively characterize the network at node levels^52^: strength (the sum of the weights of the direct relations of a node to all other nodes), closeness centrality (the inverse of the total length of all the shortest paths between the selected node and all other nodes in the network), betweenness centrality (the shortest path length connecting any two variables), expected influence (the sum of both positive and negative weights between a node and all the other nodes in the network), and degree (number of connections for each node in the network), thus defining hubs (nodes with the highest degree). The community analysis was based on the Spinglass algorithm^84^ with standard parameters (*γ* = 1, start temperature = 1, stop temperature = 0.01, cooling factor = 0.99, spins = 25). The correlation between edge weights across networks was also estimated.

## Supplementary Information

Supplementary Information.

## Data Availability

The corresponding author is responsible for submitting a competing interests statement on behalf of all authors of the paper. This statement must be included in the submitted article file.
