# Supplementary material for: A network analysis of executive functions before and after computerized cognitive training in children and adolescents
Source: Sci Rep. 2022 Aug 29;12:14660. doi: 10.1038/s41598-022-17695-x (PMC9424216; doi:10.1038/s41598-022-17695-x)
Supplement: Supplementary file 1 — Supplementary Information. [file 41598_2022_17695_MOESM1_ESM.docx]

**SUPPLEMENTARY MATERIALS**

**Table S1:** Main and interaction effects of a repeated measures ANOVA with Time (*pre-* vs *post-**training*), Training (*IC* vs *AC*) and Age (*children* vs *adolescents*) as fixed effects and with intercepts for subjects as random effects.

**Figure S1:** Levels of trained tasks reached after each training session for children (continued line) and adolescents (dashed line).

**Figure S2:** Accuracy of children’s networks.

**Figure S3:** Accuracy of adolescents’ networks.

**Table S1: Main and interaction effects of a repeated measures ANOVA with Time (*pre*- vs *post*-*training*), Training (*IC* vs *AC*) and Age (*children* vs *adolescents*) as fixed effects and with intercepts for subjects as random effects.** For all tasks, scores were derived from RTs (in s). SST = Stop Signal Task; ANT = Attention Network Test; TMT = Trail Making Test.

|  | Main effects | | | Interaction effects | | | |
| --- | --- | --- | --- | --- | --- | --- | --- |
|  | Time | Training | Age | Time*Training | Time*Age | Age*Training | Time*Training*Age |
|  | χ^2^, *p* | χ^2^, *p* | χ^2^, *p* | χ^2^, *p* | χ^2^, *p* | χ^2^, *p* | χ^2^, *p* |
| SST | 0.01, *0.91* | 0.06, *0.81* | **18.96, *1.34e-05*** | 0.07, *0.79* | 0.38, *0.54* | 0.72, *0.40* | 1.05, *0.31* |
| Stroop | 1.24, *0.27* | 0.08, *0.78* | 0.60, *0.44* | 0.08, *0.77* | 0.07, *0.80* | 0.05, *0.82* | 0.10, *0.75* |
| Simon | 1.88, *0.17* | 0.40, *0.53* | **4.16, *0.04*** | 0.00, *0.98* | 0.23, *0.63* | 0.76, *0.38* | 0.04, *0.84* |
| ANT | 0.14, *0.71* | 0.00, *0.98* | 1.74, *0.19* | 0.41, *0.52* | 2.52, *0.11* | 0.04, *0.83* | 0.24, *0.62* |
| TMT | 0.16, *0.69* | 0.19, *0.66* | **7.78, *0.01*** | 0.31, *0.58* | 1.07, *0.30* | 0.70, *0.40* | 1.05, *0.31* |
| N-back | 0.19, *0.66* | 0.08, *0.78* | 0.63, *0.43* | 0.00, *0.96* | 0.15, *0.70* | 0.54, *0.46* | 0.88, *0.35* |

| 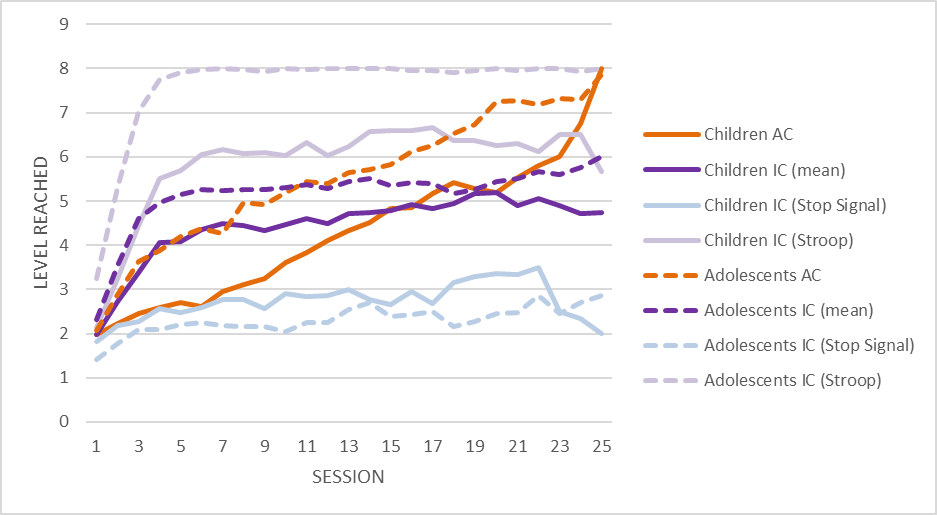 |
| --- |
|  |

**Figure S1:** **Levels of trained tasks reached after each training session for children (continued line) and adolescents (dashed line).** For IC training, the level of Stop Signal and Stroop is provided along with the average level combining the two tasks.

| **A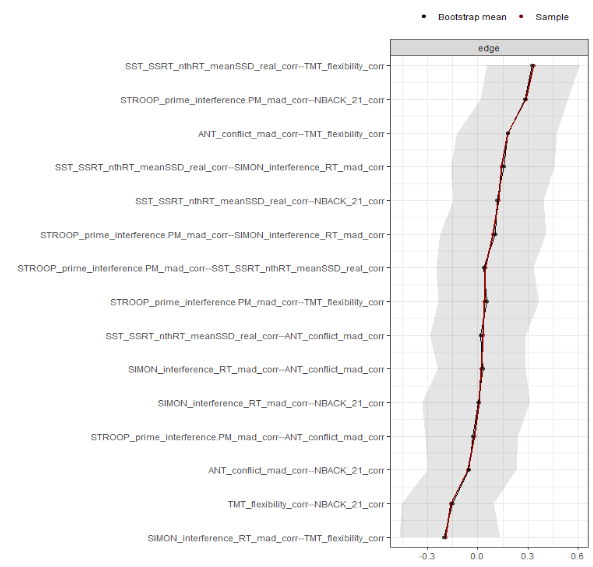** | **B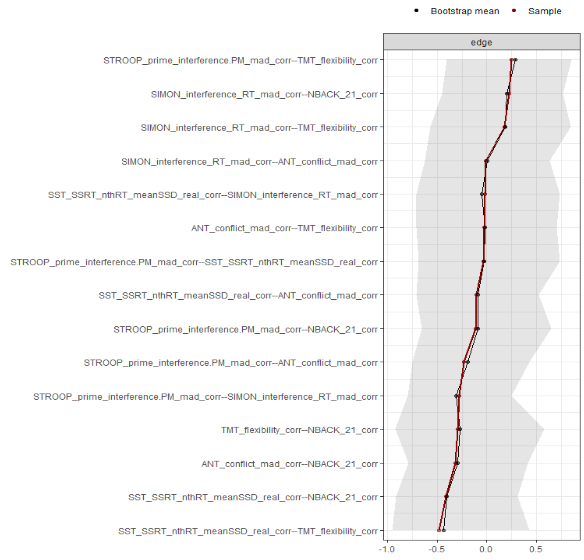** | **C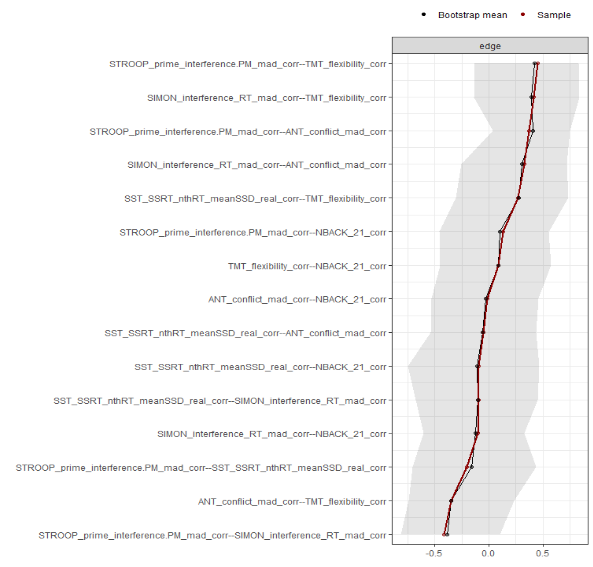** |
| --- | --- | --- |
| **D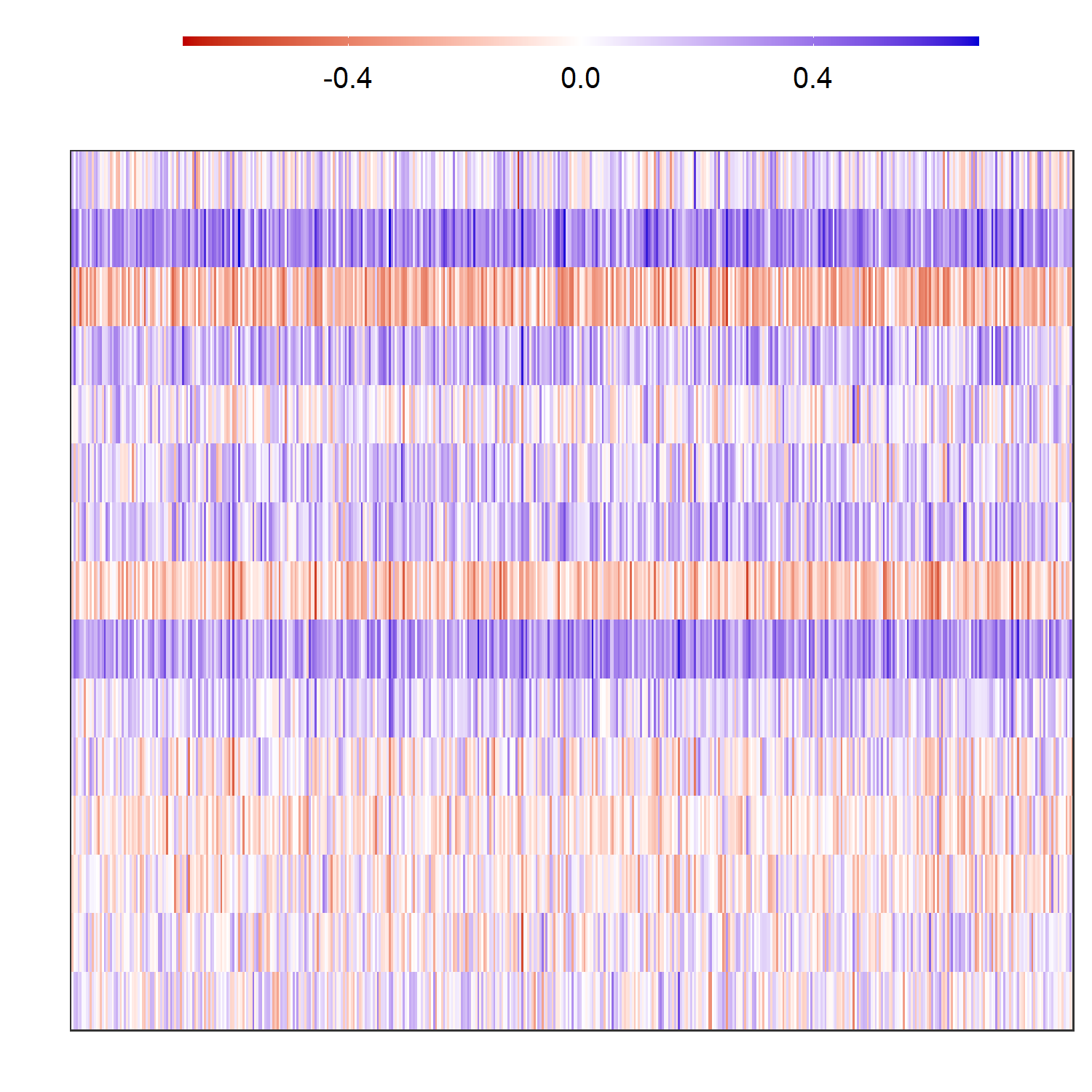** | **E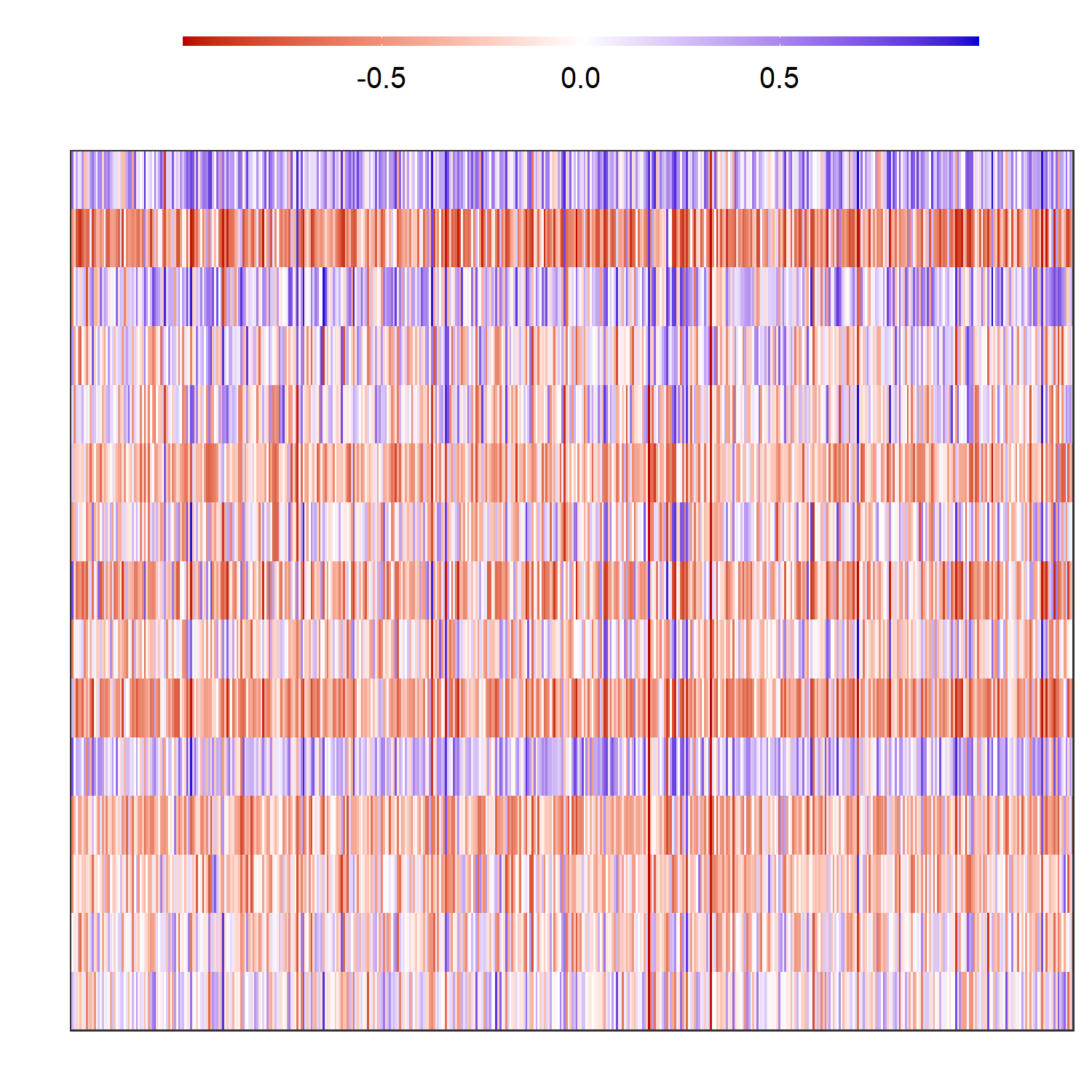** | **F**  **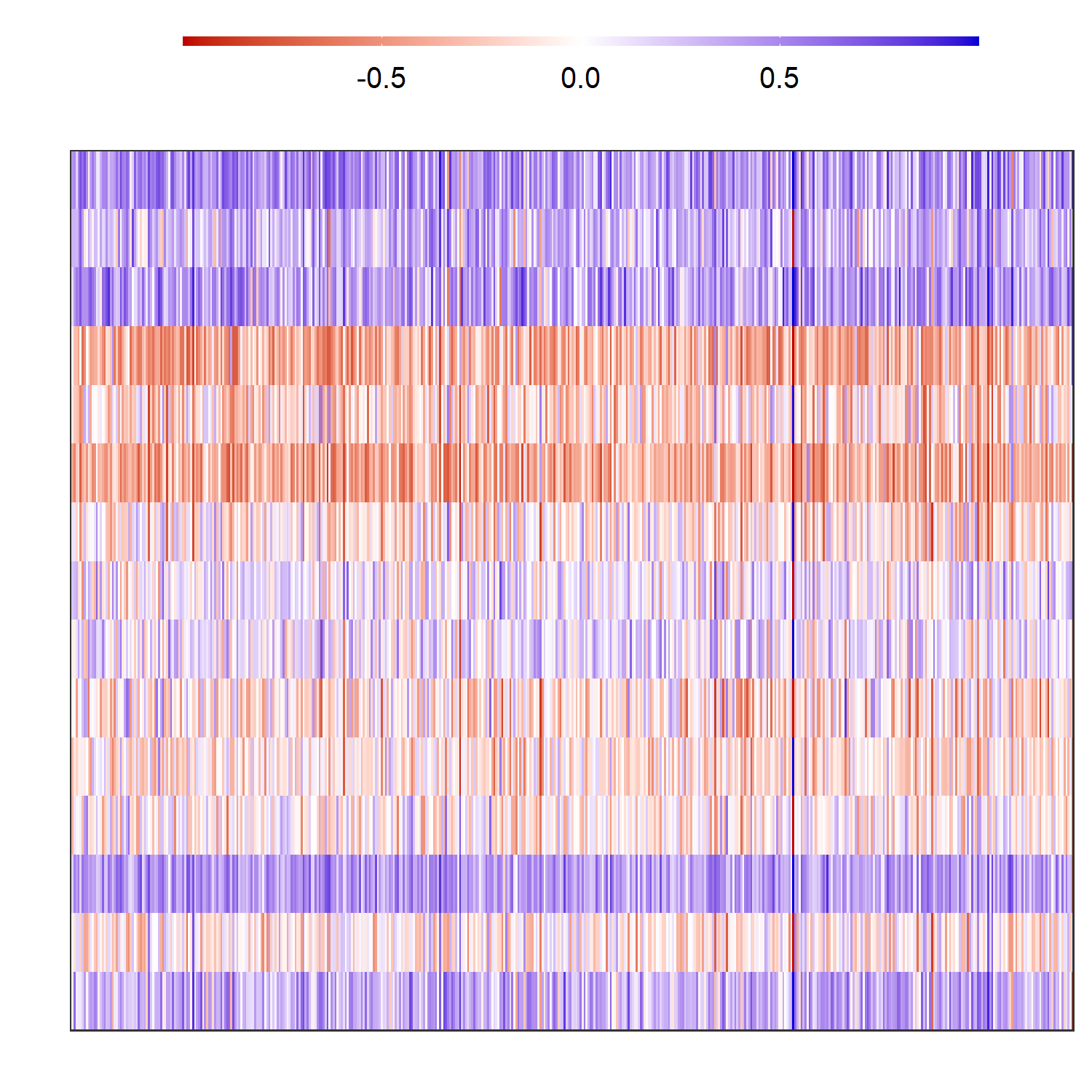** |

**Figure S2: Accuracy of children’s networks.** 95% bootstrapped CIs (nBoots = 500) for each network edge weight before (**A**), after active control training (**B**) and after inhibitory control training (**C**). Stability across bootstrap iterations (nBoots = 500) (rows: edges, columns: iterations) for the estimated networks before (**D**), after active control training (**E**) and after inhibitory control training (**F**).

| **A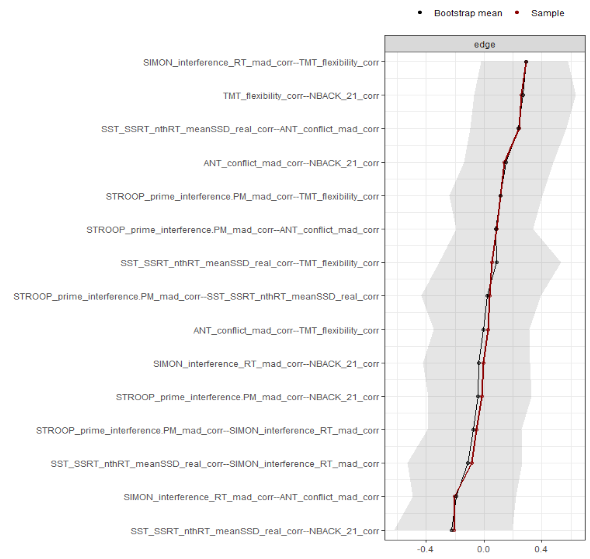** | **B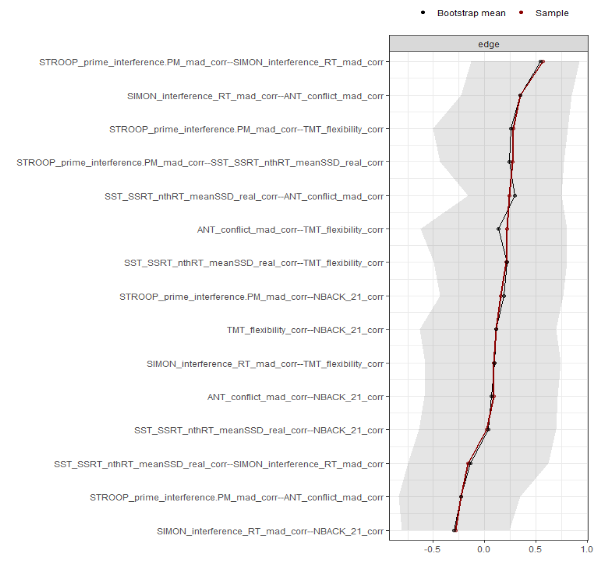** | **C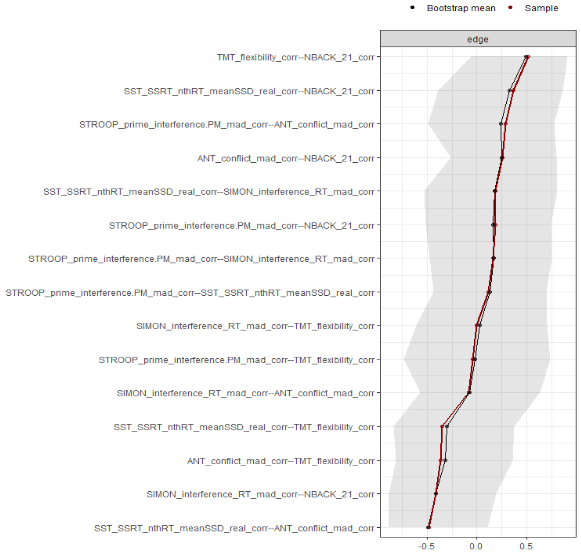** |
| --- | --- | --- |
| **D**  **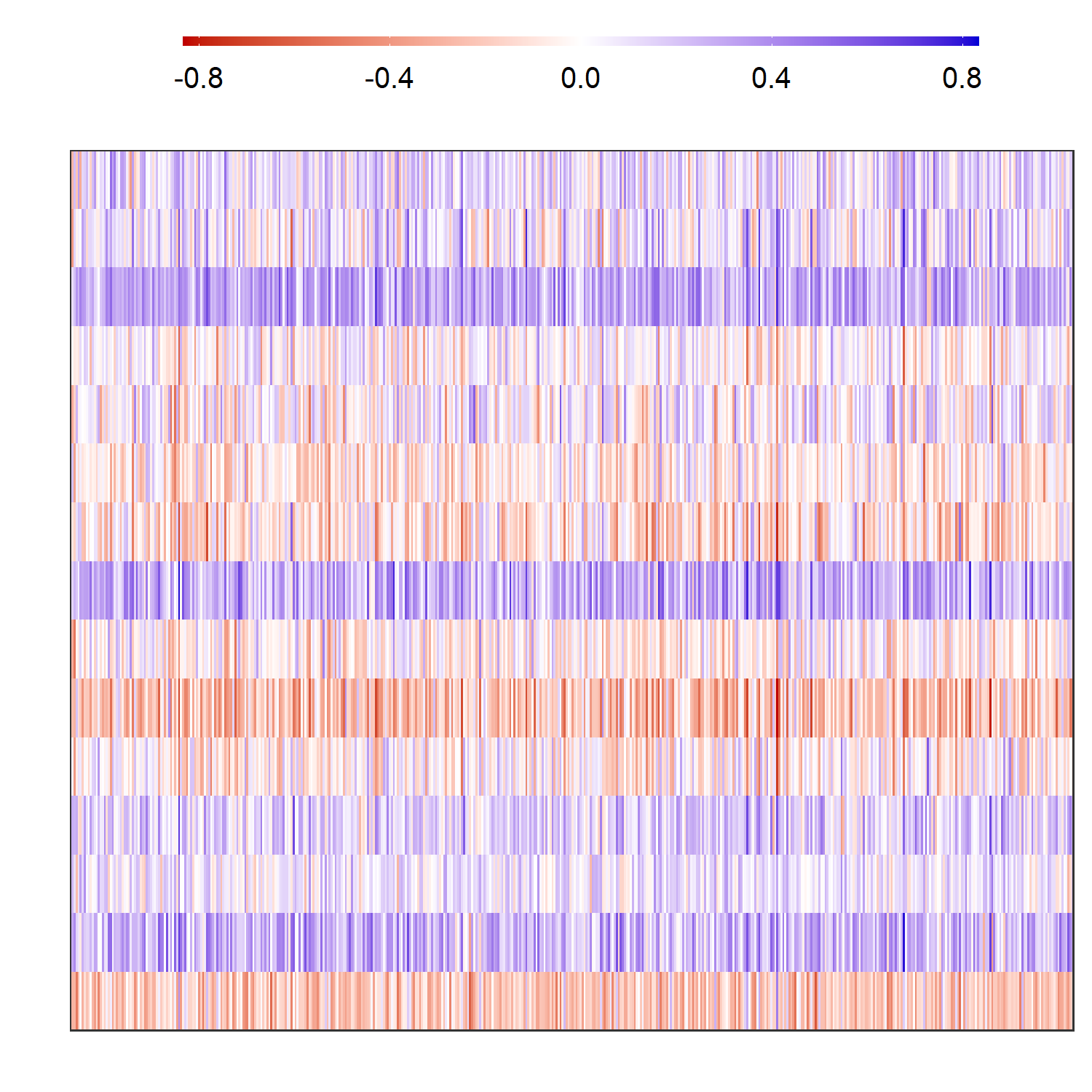** | **E**  **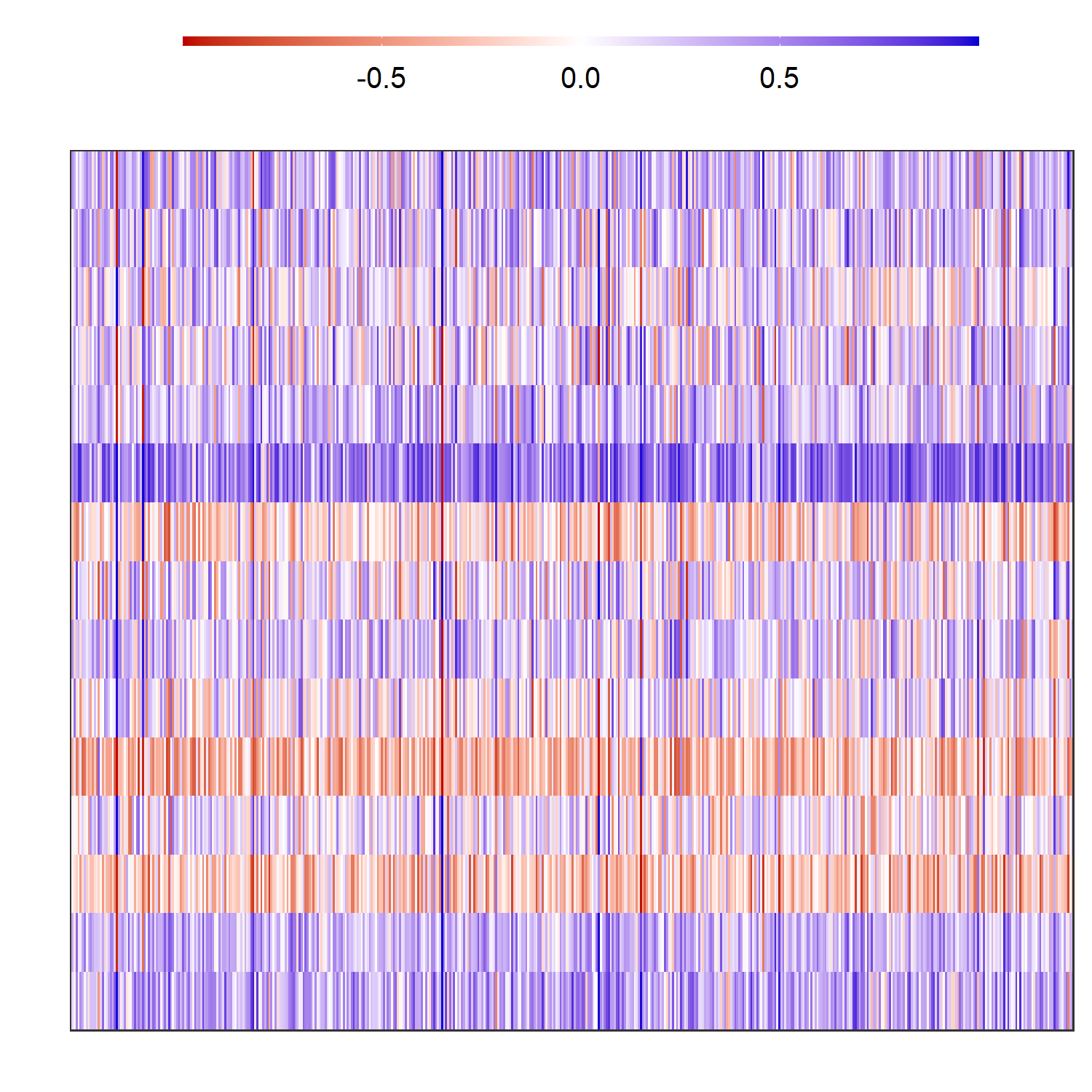** | **F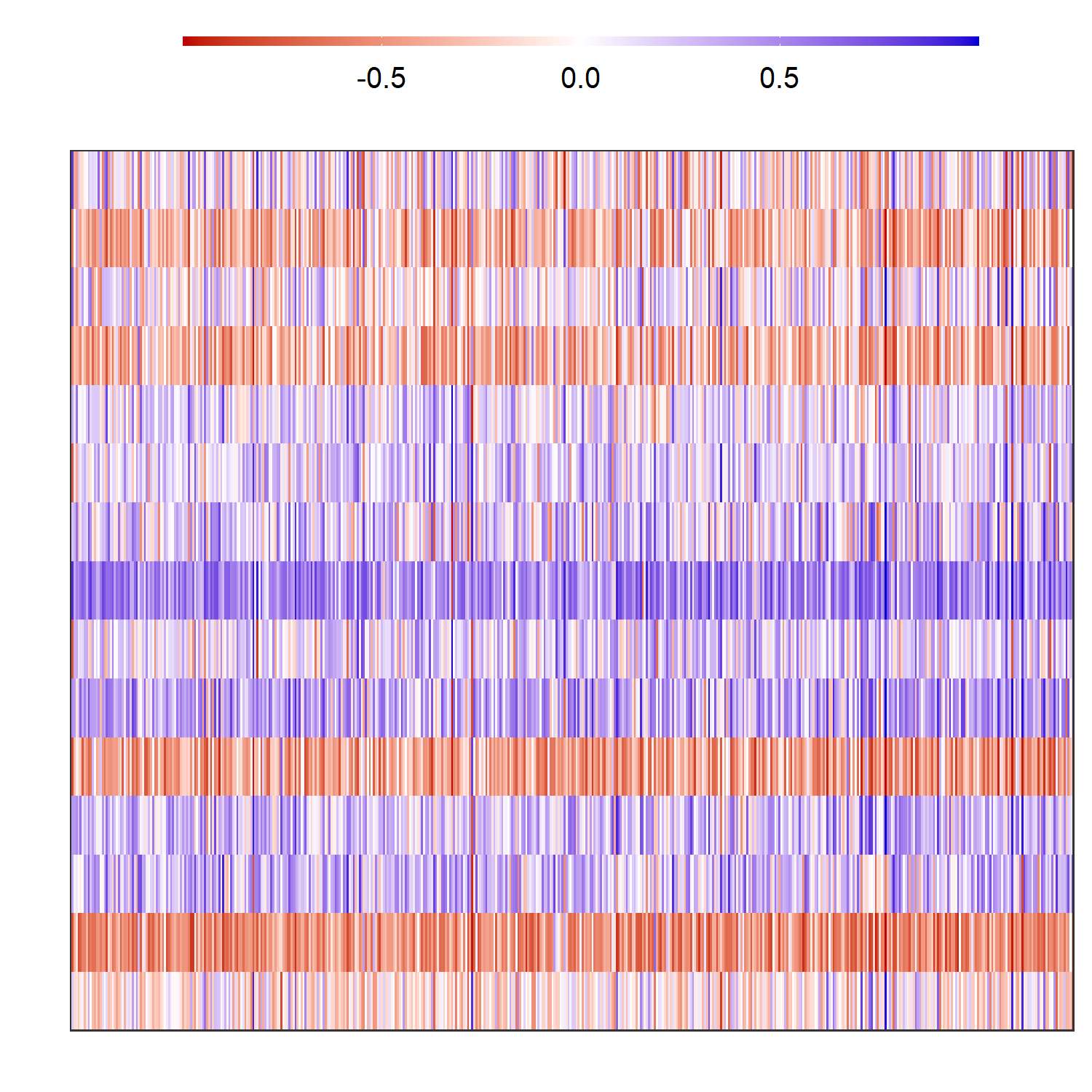** |

**Figure S3: Accuracy of adolescents’ networks.** 95% bootstrapped CIs (nBoots = 500) for each network edge weight before (**A**), after active control training (**B**) and after inhibitory control training (**C**). Stability across bootstrap iterations (nBoots = 500) (rows: edges, columns: iterations) for the estimated networks before (**D**), after active control training (**E**) and after inhibitory control training (**F**).
